# Giant facial lupus vulgaris

**DOI:** 10.1093/omcr/omac058

**Published:** 2024-04-25

**Authors:** Layla Bendaoud, Soumaya Faras, Imane Boujguenna, Hanane Raiss, Said Amal, Ouafa Hocar

**Affiliations:** Department of Dermatology, Faculty of Medicine and Pharmacy, Mohammed VI University Hospital, Cadi Ayad University, Marrakech, Morocco; Department of Dermatology, Faculty of Medicine and Pharmacy, Mohammed VI University Hospital, Cadi Ayad University, Marrakech, Morocco; Department of Dermatology, Faculty of Medicine and Pharmacy, Mohammed VI University Hospital, Cadi Ayad University, Marrakech, Morocco; Department of Dermatology, Faculty of Medicine and Pharmacy, Mohammed VI University Hospital, Cadi Ayad University, Marrakech, Morocco; Department of Dermatology, Faculty of Medicine and Pharmacy, Mohammed VI University Hospital, Cadi Ayad University, Marrakech, Morocco; Department of Dermatology, Faculty of Medicine and Pharmacy, Mohammed VI University Hospital, Cadi Ayad University, Marrakech, Morocco

A 55-year-old patient, without any particular medical history, presented to our department with red infiltrated face since last 4 years. The clinical examination revealed an extensive erythematous indurated placard covering the face, with nodular lesions on the forehead and depilation of the eyebrows (Fig. 1), without lymphadenopathy and without hepatosplenomegaly. Examination of sensibility was normal. The skin biopsy showed an epitheloid cell granuloma with caseous necrosis (Fig. 2). The bacteriological examination was negative. The tuberculin intradermal reaction was phlyctenular. Chest X-ray showed nonspecific micronodules, and sputum BK test was normal. Abdominal ultrasound showed no abnormalities. The diagnosis of lupus tuberculosis was made on the basis of the clinical and histopathologic findings. After a 9-month antituberculous therapy, the evolution was marked by the disappearance of nodular lesions and the regrowth of hair on the eyebrows with post-inflammatory pigmentation.

**Figure 1:**
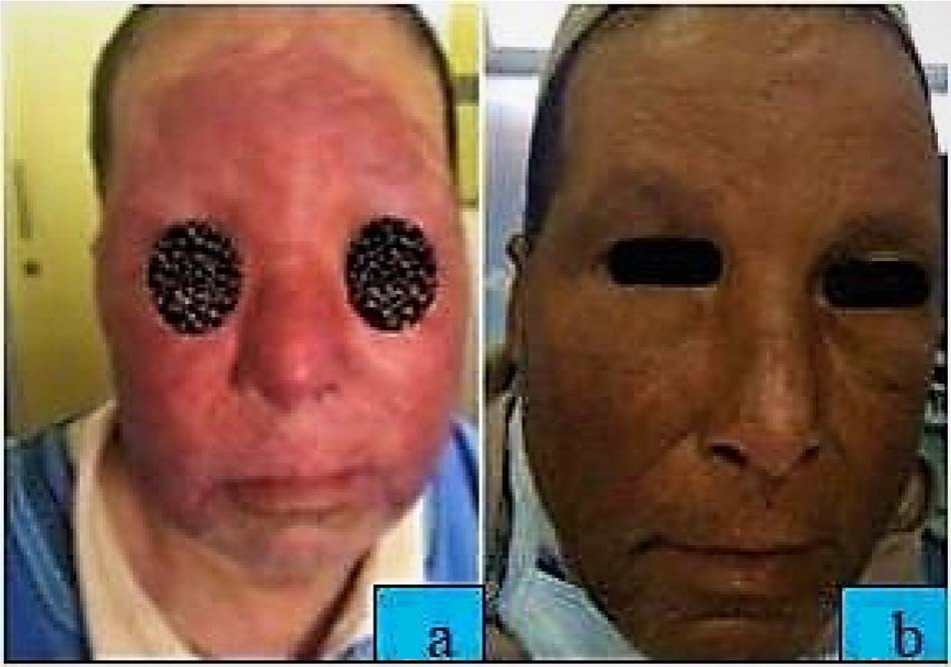
:(**a**) Red infiltrated plaque on face; (**b**) Improvement after antituberculous therapy.

**Figure 2:**
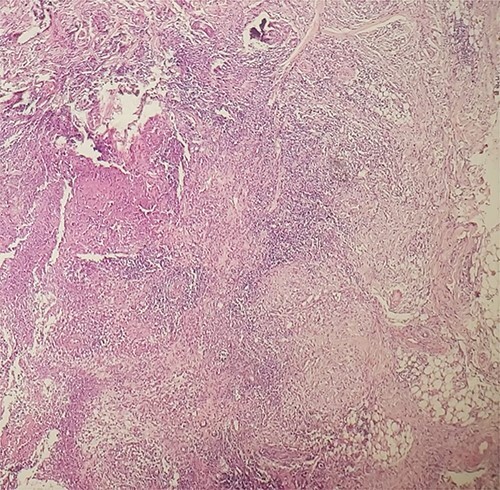
Tuberculoid granuloma with caseous necrosis.

Cutaneous tuberculosis (TB) results from skin infection with *Mycobacterium tuberculosis*. It is an uncommon form of extrapulmonary tuberculosis. Lupus vulgaris is the most common form of TB [1], and it is usually located on the head and neck region. Clinically, lupus vulgaris is characterized by a macule or papule, with a brownish-red color, that coalesces to form larger plaques [2]. Histopathological study shows tuberculoid granulomas [3]. The diagnosis of lupus vulgaris is usually based on clinical and histopathological findings. The treatment consists of 2 month of Rifampicin, Isoniazid, Ethambutol and Pyrazinamide, then 4 months of Rifampicin and Isoniazid.

## CONFLICT OF INTEREST STATEMENT

The authors declare no conflict of interest.

## FUNDING

None declared.

## ETHICAL APPROVAL

No ethics committee approval is required for case reports at our institution.

## CONSENT

The patient has given written informed consent for the publication of this manuscript and images.

## GUARANTOR

Layla Bendaoud.
